# Nursery Treatment of Infants, Submitted to Prince Albert

**Published:** 1843-04

**Authors:** Joshua Waddington

**Affiliations:** Consulting Surgeon of the Royal Sea-Bathing Infantry


*Nursery treatment of Infants, submitted to Prince Albert.* By Joshua Waddington, M. R. C. S., consulting Surgeon of the Royal Sea-Bathing Infantry.—No other kind of milk to be given to an infant in addition to the milk of the mother or wet-nurse.

The less rocking the better.

When asleep to be laid upon its *right* side.

The best food is "Lemann's biscuit-powder," soaked for 12 hours in cold spring water, then *boiled* for half an hour, not simmered, or it will turn sour. Very little sugar to be added to the food, and then only at the time *when given.*

Sweets of every kind, are most injurious, producing acidity, flatulency, and indigestion, sores in the mouth, and disordered secretions.

An infant will take medicine the more readily if made lukewarm in a cup placed in hot-water, adding a very little sugar *when taken.*

The warm-bath (at ninety-four degrees of heat, *not less,* for *ten* minutes, every other night) is a valuable remedy in many cases of habitual sickness or constipation.

"Soothing syrup," sedatives, and anodynes, of every kind, are most prejudicial. They stop the secretions. A very small dose of laudanum given to an infant may produce coma and death.

When an infant is weaned, which is generally advisable at the age of nine months, it is of the utmost importance that it be fed with the milk of *one cow,* and *one only,* (a milch-cow), mixed with "Lemann's biscuit powder" (prepared as before directed) and *very little sugar.*

Boiled bread-pudding forms a light and nutritious dinner, made with stale bread, hot milk, an egg, and very little sugar.

When an infant is twelve months of age, bread and milk should be given every night and morning: stale bread toasted, soaked in a little hot-water, and then the milk (of *one cow*) added *cold.*

Solid meat is not generally required until an infant is fifteen months of age, and then to be given sparingly, and cut very fine. Roasted mutton, or broiled mutton-chop (without fat), is the best meat; next to that, tender *lean* beef or lamb; then fowl, which is better than chicken; no pork or veal; no pastry; no cheese; *the less butter the better.*

An infant should not be put upon its feet soon, especially while *teething,* or *indisposed.*

*Avoid over-feeding* at all times, more particularly when *teething.* It is very likely to produce indigestion and disordered secretions, the usual *primary causes* of convulsions, various eruptive complaints, and inflammatory affections of the head, throat, and chest.

*London Lancet,* December 24, 1842.

